# A Periodic Case of Maxillo-Nasal Dysplasia or Binder Syndrome Successfully Operated With Bilateral Le Fort II Osteotomy With Distraction Osteogenesis

**DOI:** 10.7759/cureus.29706

**Published:** 2022-09-28

**Authors:** Switi Jawade, Vaishnavi V Kantode, Mayur B Wanjari, Abhyuday Meghe, Dr. Ranjana Sharma, Pratibha Wankhede, Deeplata M Mendhe

**Affiliations:** 1 Department of Nursing, Florence Nightingale Training College of Nursing, Datta Meghe Institute of Medical Sciences, Wardha, IND; 2 Department of Medical Surgical Nursing, Smt. Radhikabai Meghe Memorial College of Nursing, Datta Meghe Institute of Medical Sciences, Wardha, IND; 3 Research Scientist, Jawaharlal Nehru Medical College, Datta Meghe Institute of Medical Sciences, Wardha, IND; 4 Medicine, Acharya Vinoba Bhave Rural Hospital, Jawaharlal Nehru Medical College, Datta Meghe Institute of Medical Sciences, Wardha, IND; 5 Department of Community Health Nursing, Smt. Radhikabai Meghe Memorial college of Nursing, Datta Meghe Institute of Medical Sciences, Wardha, IND

**Keywords:** naso-maxillary hypoplasia, distraction osteogenesis, bilateral lefort ii osteotomy, maxillo-nasal dysplasia, binder's syndrome

## Abstract

Binder syndrome (BS) is an uncommon congenital disorder affecting the face. The condition, which also goes by the names naso-maxillary hypoplasia (NMH) and maxilla-facial dysplasia (MFD), causes the central face to develop inward and may also affect the upper jaw and the nose. A 19-year-old male with a known case of BS presented with a complaint of poor esthetics since birth. Previously, the patient was admitted to a private hospital where he was operated on for cleft lip and palate in the years 2003 and 2005. In 2017, he visited the dental clinic where the orthodontic treatment started for poor esthetics, and then he was referred to the oral surgery ward for surgical intervention. For about five years, he has been undergoing orthodontic treatment. A physical examination of the oral cavity was done and the physician suggested a CT scan of the brain. Recently, the patient underwent bilateral Le Fort II osteotomy with distraction osteogenesis under general anesthesia which repaired the patient's esthetics.

## Introduction

The term "Binder type naso-maxillary dysplasia" was first mentioned in medical literature in 1882 after identifying the unique characteristics of just one patient [1]. Binder syndrome (BS), also known as maxillo-nasal dysplasia and naso-maxillary hypoplasia (NMH), is a rare developmental defect that primarily affects the anterior portion of the maxilla and nasal complex [2]. BS affects men and women equally. The actual incidence or prevalence is still unknown but it approximately affects one live newborn out of every 10,000 births [3]. Several studies have identified various environmental causes that may be likened to BS-type naso-maxillary dysplasia including birth trauma, vitamin K deficiency, exposure of a developing infant to an anti-seizure drug known as phenytoin or to an anti-blood-clotting (anticoagulant) drug known as warfarin [2].

## Case presentation

**Figure 1:**
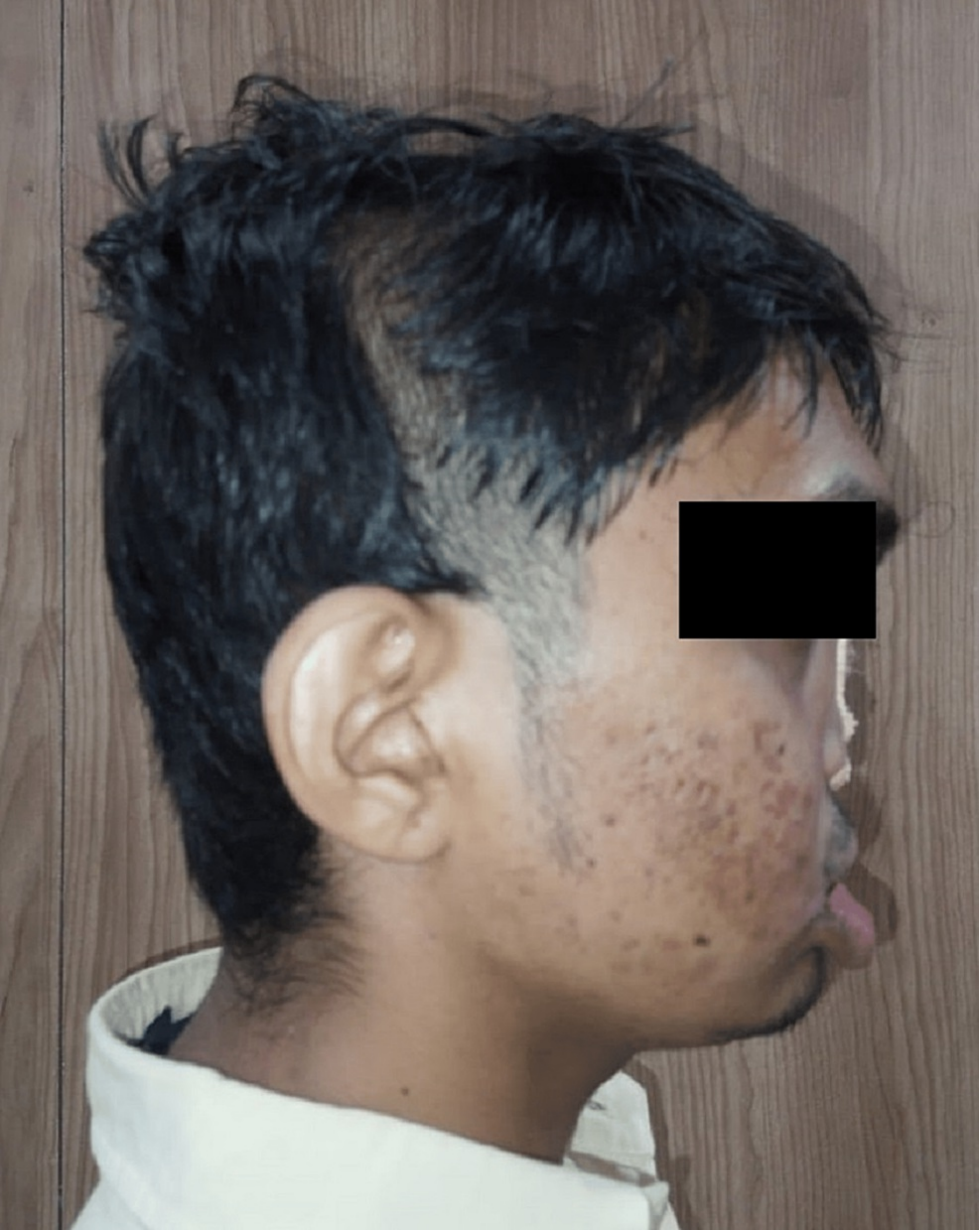
Flattened Nose and Upper Lips

A 19-year-old male with a known case of BS came with a complaint of poor esthetics since birth (Figure 1). In the year 2003, the patient underwent cleft lip surgery when he was eight months old and also underwent cleft palate surgery in the year 2005 in a private hospital. In 2017, he visited the dental clinic where the orthodontic treatment started, he was then referred to the oral surgery department for further management. He had a previous history of hospitalization for the same problem and a history of blood transfusion during the first operation. For approximately five years, he has been undergoing orthodontic treatment.

**Figure 2:**
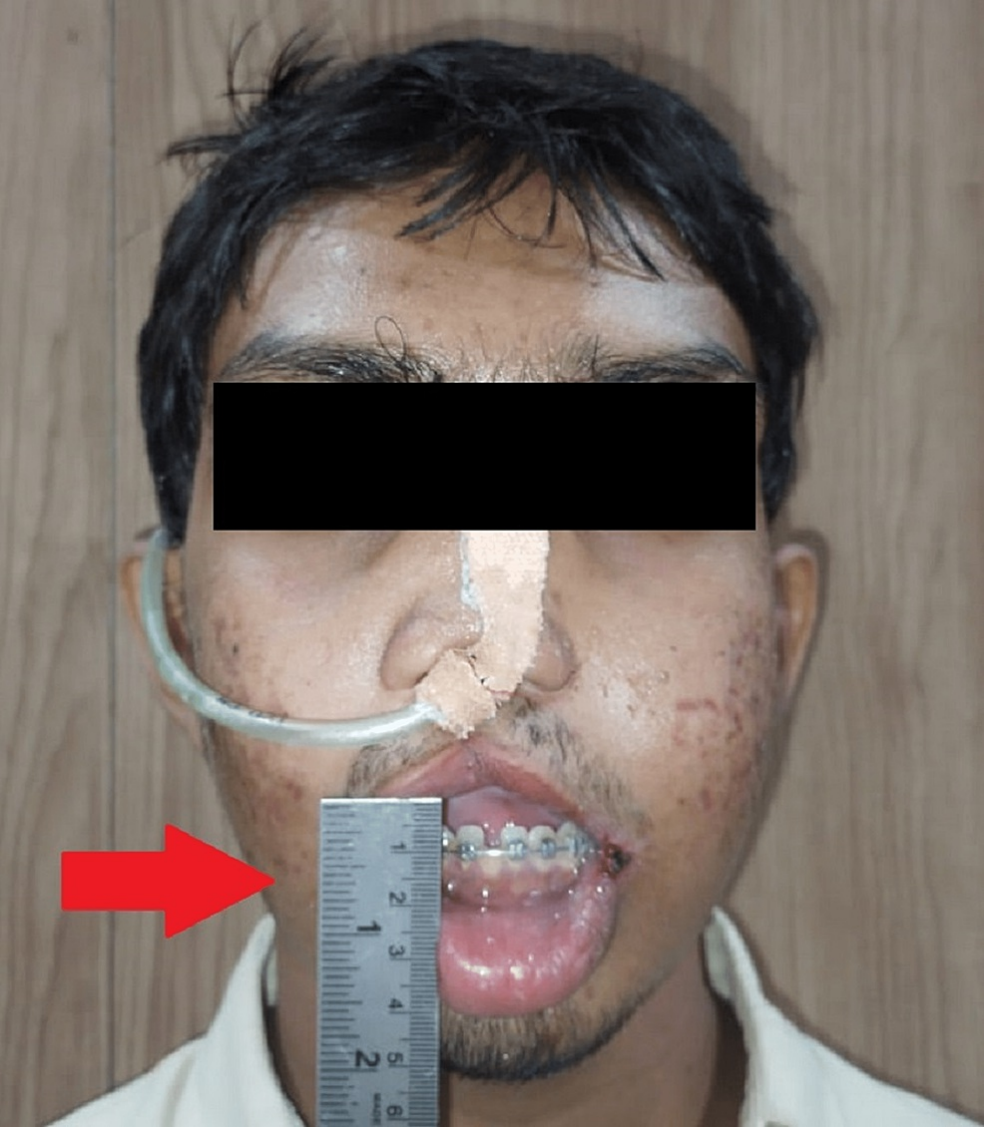
Grossly Asymmetrical Face, Incompetent Lips, Depressed Nasal Bridge, and Horizontal Nostrils with Mouth Opening 40 mm

During the extra-oral examination, the face looks grossly asymmetrical due to a retrognathic maxilla. Lips were incompetent (Figure 2). The temporomandibular joint was smooth and synchronous with the presence of a clicking sound. During the intraoral examination, the mouth opening was adequate (40 mm), the uvula was bifid, and orthodontic brackets and wire were seen from the region 13-16, 23-26, 31-36, 41-46. Alveolar fistula was present over the 11 region. 

CT of the brain revealed a bony defect in the form of a hypoplastic nasal bone and nasal septum with an unfused hard palate in the midline with surgical wiring in situ. Dysgenesis of the anterior aspect of the corpus callosum was evident. The patient has undergone bilateral Le Fort II osteotomy with distraction osteogenesis under general anesthesia. After surgery, the patient was shifted to the oral surgery unit for monitoring and kept nil by mouth. Later, he was treated with analgesics (injection neomol 1 gm twice a day, injection tramadol 50 mg twice a day), antibiotics (injection augmentin 1.2 gm twice a day, injection metro 500 mg twice a day), and intravenous fluids. The patient's prognosis was good and during the follow-up period, no obvious anomalies were detected. 

## Discussion

The exact cause of the syndrome is yet unknown. Patients with BS typically have midfacial hypoplasia, resulting in a flat nose, flattened tip and alar wings, half-moon-shaped nostrils, a short columella, an acute nasolabial angle, and a frontonasal angle of nearly 180 degrees, which eventually leads to a concave midfacial profile. Depending on the severity of the illness, most patients exhibit a few or all of the characteristics listed [4]. Based on typical clinical and radiologic features, BS is diagnosed [5]. Patients require surgery and orthodontic treatment (braces) because of their clinical appearance [6].

Depending on the patient's age, there are different approaches to treating BS. The placement of endonasal and gastric tubes may be necessary for the newborn due to respiratory and digestive issues brought on by the abnormality. Children with BS should have orthodontic treatment as soon as indicated to correct their occlusal issues and orofacial dysfunctions. A patient may undergo augmentation of the premaxilla surgery once they reach adulthood [7]. Orthognathic, otolaryngologic, plastic, or reconstructive surgery are all possible treatments for BS. A Le Fort I and/or II maxillary osteotomy may be required to progress the hypoplastic part of the bony skeleton [8]. In order to treat BS, osteotomies and cartilage or bone grafts are routinely employed [9].

Depending on the seriousness of the disease, Le Fort I, Le Fort II, or Le Fort III can be used to treat BS. The traditional Le Fort II osteotomy is the most frequently recommended for treating this condition. Oral and maxillofacial surgeons very commonly execute the original classic Le Fort II operation since most patients with mid-face deficiencies need more lateral infraorbital rim and zygoma augmentation. Therefore, quadrangular Le Fort II is typically the recommended method [10]. The Le Fort I osteotomy is frequently used to treat maxillomandibular abnormalities and malocclusions [11]. If no additional problem is associated with BS, then the prognosis is excellent [12].

## Conclusions

BS is a rare congenital disorder that is also known as maxillo-nasal dysplasia and NMH. It primarily affects the anterior portion of the maxilla and nasal complex. The cause is still unknown. Patients who present with this syndrome have unique facial appearances. The only surgical choices available are maxillary advancement and nasal dorsum and apex reconstruction. Following orthodontic rehabilitation, the management entails nasal and maxillary correction. A treatment strategy developed in cooperation between orthodontists and ENT surgeons must be applied to patients with BS. The prognosis of BS varies depending on the various accompanying features it presents, which is a vital feature to comprehend it. An appropriate differential diagnosis needs to be made after the diagnosis.
